# Clinical characteristics and factors influencing hospitalization in pediatric patients with foreign body aspiration: a comprehensive analysis in an emergency department

**DOI:** 10.1186/s12245-025-00923-2

**Published:** 2025-07-01

**Authors:** Piyadarat Asawasakulchokedee, Rattapon Uppala, Phanthila Sitthikarnkha, Sirapoom Niamsanit, Leelawadee Techasatian, Suchaorn Saengnipanthkul, Pornthep Kasemsiri

**Affiliations:** 1 Department of Pediatrics, Faculty of Medicine, Khon Kaen University, Khon Kaen, 40002 Thailand; 2 Department of Otorhinolaryngology, Faculty of Medicine, Khon Kaen University, Khon Kaen, 40002 Thailand

**Keywords:** Foreign body aspiration, Pediatric emergency, Rigid bronchoscopy, Hospitalization, Airway management

## Abstract

**Background:**

Foreign body aspiration (FBA) in children is a potentially life-threatening event, especially in those under 3 years of age. This study aimed to identify clinical factors associated with hospitalization in pediatric FBA and to highlight innovative retrieval methods used in a tertiary care setting.

**Methods:**

A retrospective analysis was conducted of pediatric patients (< 18 years) presenting with confirmed airway FBA between January 2015 and September 2023 at Srinagarind Hospital, Khon Kaen University. Demographic information, clinical characteristics, and procedural data were collected. Univariate and multivariate logistic regression analyses identified predictors of hospitalization.

**Results:**

Among 297 pediatric patients with FBA, 39 (13.1%) required hospitalization. Hospitalized children were younger (median age: 3 years; interquartile range [IQR] 2–7) compared with non-hospitalized children (median age: 4 years; IQR 3–7, *p* = 0.018). FBA involving the larynx-trachea-bronchus significantly increased the likelihood of admission (adjusted odds ratio [AOR] = 22.0; 95% confidence interval [CI]: 6.64–72.63; *p* < 0.001). Delayed presentation to the emergency department more than two hours after onset was also associated with hospitalization (OR = 2.77; 95% CI: 1.08–7.09; *p* = 0.033). Although rigid bronchoscopy remained the mainstay treatment, flexible bronchoscopy was successfully utilized in nine cases. Notably, a novel technique using a 3 mm gold-plated neodymium magnet under fluoroscopic guidance enabled the safe retrieval of distal metallic foreign bodies. Complete removal of the aspirated objects was achieved in all patients; however, one patient died following prolonged hypoxia prior to hospital arrival.

**Conclusions:**

Younger age, delayed emergency department presentation, and location of the foreign body in the larynx-trachea-bronchus are significant predictors of hospitalization in pediatric FBA. Rigid bronchoscopy remains the primary management strategy, while flexible bronchoscopy and innovative retrieval methods, including magnet-assisted approaches, offer effective alternatives in specific scenarios. Emphasizing early diagnosis and rapid intervention is essential to reduce complications and improve outcomes in children with FBA.

## Introduction

Foreign body aspiration (FBA) is a frequent emergency cause of acute respiratory distress in children and can be life-threatening if not promptly diagnosed and treated [1]. Globally, children under 3 years are at especially high risk, predominantly because of their developmental tendency to explore objects orally [2]​. Indeed, 75–90% of FBA cases occur in children under 6 years of age​ [3, 4].

Clinical manifestations of FBA vary, influenced by the size, type, and location of the aspirated object. Common symptoms include coughing, dyspnea, and wheezing [5, 6]. Most cases result in mild outcomes, but delayed diagnosis can precipitate severe complications such as pneumonia, atelectasis, and in rare instances, death [7, 8]. Diagnostic delays may stem from limited clinical expertise, unavailability of bronchoscopy facilities, and delayed access to medical attention [9–11].

Although rigid bronchoscopy has traditionally been considered the gold standard for both diagnosis and treatment of FBA, the potential for unnecessary invasive procedures has prompted the increasing use of flexible bronchoscopy, which can serve both diagnostic and therapeutic roles in select cases [12–14]. Much of the literature focuses on single-center experiences or specific pediatric cohorts, leaving gaps in the understanding of broader epidemiological patterns and risk factors for hospitalization related to FBA.

This study aimed to address these gaps by analyzing clinical characteristics and factors associated with hospitalization in pediatric FBA. Utilizing data from a tertiary care center, we sought to identify demographic, clinical, and anatomical predictors of hospitalization. Additionally, we describe novel retrieval methods, including a magnet-assisted approach, to inform future management strategies and improve treatment outcomes for children worldwide.

## Materials and methods

### Study design and participants

This retrospective study examines children under 18 years diagnosed with airway foreign body aspiration and treated in the emergency department (ED) of Srinagarind Hospital, Khon Kaen University, between January 1, 2015, and September 30, 2023. The study includes all eligible cases within this period, using medical record data to identify etiological and predictive factors, as well as treatment outcomes. Inclusion criteria are children under 18 years with a confirmed diagnosis of airway foreign body aspiration, the exclusion criteria, including non-airway foreign body cases (such as esophageal foreign body) or incomplete medical records. The Strengthening the Reporting of Observational Studies in Epidemiology (STROBE) checklist was used to report the results of this study [15].

### Data collection

Demographic data included age (years, months), gender, and circumstances of the aspiration (e.g., whether it occurred during a meal. The time interval from onset of symptoms to ED presentation was categorized as < 1 h, 1–2 h, or > 2 h. The anatomical location of the foreign body (e.g., nasal cavity, pharynx, or larynx-trachea-bronchus) was also recorded, alongside the method of removal (e.g., rigid versus flexible bronchoscopy) and the final disposition (hospitalization versus discharge from the ED). The models and sizes of bronchoscopes used in out setting including of Karl Storz^®^ Rigid Bronchoscope 3.5, 3.7, 4.0 mm and flexible Bronchoscope Olympus^®^ 2.8, 4.2, 4.9 mm.

### Statistical analyses

Data were analyzed using STATA software version 10 (StataCorp, College Station, TX). Categorical variables are presented as frequencies and percentages, while continuous variables are summarized as medians with interquartile ranges (IQR) or means with standard deviations (SD), depending on data distribution. Between-group comparisons (hospitalized vs. non-hospitalized) were made using the Chi-square test or Fisher’s exact test for categorical variables and the Mann-Whitney U test for non-normally distributed continuous variables.

Variables with a *p*-value < 0.2 in univariate analyses were included in a multivariate logistic regression to adjust for potential confounders, with results reported as odds ratios (OR) and 95% confidence intervals (CI). A *p*-value < 0.05 was considered statistically significant.

### Ethics approval

This study was approved by the Khon Kaen University Ethical Committee (KKUEC), approval number HE661484, in accordance with the Declaration of Helsinki and Good Clinical Practice guidelines. The written informed consent to participate in this study was waived by the Institutional Review Board of KKUEC as per Khon Kaen University’s Announcement No. 2179/2563, the process of obtaining subjects’ consent was waive as this is a study of existing data or biological specimens without further prospective data collection from or direct interactions with the subjects. The written inform consent for publication was obtained from the patient’s legal guardian for the publication of any potentially identifiable images or data included in this article.

## Results

A total of 297 pediatric patients with confirmed FBA were identified. Of these, 258 (86.9%) were discharged from the ED, and 39 (13.1%) were hospitalized for further management or observation. Table 1 presents demographic and clinical characteristics.

**Table 1:** Demographic and clinical features of pediatric patients with foreign body aspiration in emergency departments

	No hospitalization (*n* = 258)	Hospitalization (*n* = 39)	*p*-value
Age (year)			
Median (interquartile range; IQR)	4(3–7)	3(2–7)	0.018^a^
<3	40 (15.5)	16 (41.0)	< 0.001^b^
3-<5	107 (41.5)	8 (20.5)	
5–7	111 (43.0)	15 (38.5)	
Gender			0.989^c^
Male	126(48.8)	19(48.7)	
Female	132(51.2)	20(51.3)	
Meal-related event			0.555^c^
No	173 (67.0)	28 (71.8)	
Yes	85 (32.9)	11 (28.2)	
Location of the event			0.865^b^
Home	98(38.0)	16(41.0)	
Kindergarten	75(29.1)	12(30.8)	
Outdoor activities	85(32.9)	11(28.2)	
Location of foreign bodies			< 0.001^b^
Nasal cavity	134 (51.9)	8 (20.5)	
Pharynx	110 (42.6)	6 (15.4)	
Larynx-trachea-bronchus	14 (5.5)	25 (64.1)	
Onset prior to ED visit (hour)			0.079^b^
Less than 1	91 (45.5)	7 (25.9)	
1–2	34 (17.0)	4 (14.8)	
More than 2	75 (37.5)	16 (59.3)	

^a^Mann-Whitney test,^b^Fisher Exact test,^c^Chi square test

### Demographic and clinical features

Hospitalized patients were younger, with a median age of 3 years (IQR 2–7) compared to 4 years (IQR 3–7) in non-hospitalized patients (*p* = 0.018). No significant differences in gender distribution were observed (*p* = 0.989). Although meal-related events appeared more common in the hospitalized group, this association was not statistically significant after adjusting for confounders. Foreign body location was a critical factor: larynx-trachea-bronchus involvement was significantly higher in hospitalized patients (64.1%) than in non-hospitalized patients (5.4%; *p* < 0.001). Delayed presentation (> 2 h) was more common in hospitalized patients (59.3% vs. 37.5%), but this factor reached statistical significance only in the univariate analysis (*p* = 0.033).

### Predictors of hospitalization

Table 2 shows univariate and multivariate logistic regression analyses for factors associated with hospitalization. Younger age (< 3 years) was significant in the univariate model (OR = 2.96, 95% CI 1.34–6.53; *p* = 0.007) but did not remain significant after adjustment (AOR = 2.78, 95% CI 0.86–8.98; *p* = 0.086). Foreign body location in the larynx-trachea-bronchus persisted as a strong predictor of hospitalization (AOR = 22.0, 95% CI 6.64–72.63; *p* < 0.001). Delayed ED presentation (> 2 h) significantly increased odds of hospitalization (OR = 2.77, 95% CI 1.08–7.09; *p* = 0.033) (Table 2).

**Table 2:** Factor associated with hospitalization in pediatric patients with foreign body aspiration in emergency departments

	Univariate OR (95%CI)	*p*-value	Multivariate AOR (95%CI)	*p*-value
Age (year)				
<3	2.96 (1.34–6.53)	0.007	2.78 (0.86–8.98)	0.086
3-<5	0.55 (0.22–1.35)	0.197	1.03 (0.29–3.65)	0.960
5–7	1		1	
Gender				
Male	1		1	
Female	1.00 (0.51–1.97)	0.989	0.86 (0.33–2.25)	0.772
Meal-related event				
No	1		1	
Yes	0.79 (0.37–1.68)	0.370	0.90 (0.26–3.00)	0.864
Location of foreign bodies				
Nasal cavity	1		1	
Pharynx	0.91 (0.31–2.71)	0.871	1.41 (0.39–5.10)	0.59
Larynx-trachea-bronchus	29.91 (11.36–78.74)	< 0.001	22.0 (6.64–72.63)	< 0.001
Onset prior to ED visit (hour)				
Less than 1	1		1	
1–2	1.53 (0.42–5.56)	0.519	3.05 (0.72–12.90)	0.120
More than 2	2.77 (1.08–7.09)	0.033	2.96 (0.96–9.05)	0.057

### Management and outcomes

All nasal and pharyngeal foreign bodies were successfully removed using specialized forceps (e.g., alligator or bayonet forceps). Rigid bronchoscopy was employed under general anaesthesia in 31 cases involving the larynx-trachea-bronchus. Nine patients required flexible bronchoscopy under general anaesthesia (laryngeal mask airway) for foreign bodies lodged in a distal bronchus. These were retrieved by various methods: Grasping forceps (4 cases), Retrieval basket (2 cases), Fogarty balloon catheter, used to pull the foreign body proximally for subsequent extraction with rigid bronchoscopy (1 case), Magnet-assisted retrieval of distal metallic foreign bodies (2 cases).

**Fig. 1:**
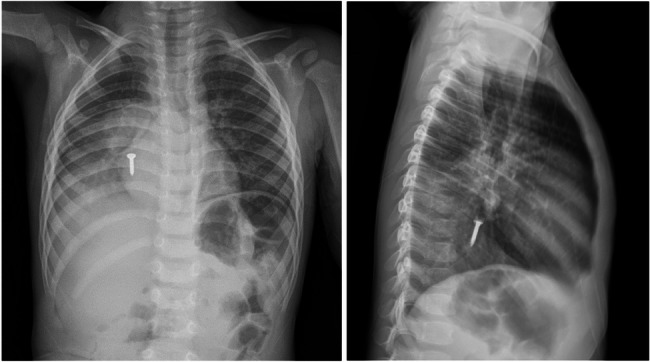
Chest X-ray (upright and left lateral) showing distal foreign body obstruction of a tiny nail in a 2-year-old boy

To manage distal metallic foreign bodies as shown in Chest X-ray (Fig. 1), we initially attempted removal with standard devices such as zero-tip baskets, polypectomy baskets, and snares; however, these proved ineffective due to the object’s shape and edges. Consequently, we employed a novel technique using a 3-mm gold-plated neodymium magnet (originally designed for endoscopic procedures) secured to bronchoscopic grasping forceps with silk 3.0. Under real-time fluoroscopic guidance, the magnet was navigated to attach to the metallic foreign body. The procedure was completed successfully without complications, and the patient was discharged the following day. To our knowledge, this is the first reported utilization of this combined fluoroscopic and magnet-assisted approach for a distal bronchial foreign body.

Complete removal of the aspirated object was achieved in all cases. One patient, unfortunately, suffered prolonged hypoxia prior to hospital arrival. Although the foreign body was removed, the child developed brain death and died subsequently.

## Discussion

These findings underscore critical clinical factors affecting hospital admission in pediatric patients with FBA. In particular, delayed presentation to the ED and the anatomical site of the foreign body emerged as significant predictors of hospitalization in this study. The median age of hospitalized patients in this study was 3 years, aligning with earlier research indicating that children under 3 years face the highest risk of FBA-related, young children are especially vulnerable to FBA owing to their developmental tendencies, smaller airway size, and propensity for oral exploration [16–18].

In this study, foreign bodies lodged in the larynx, trachea, or bronchus posed the greatest association of hospitalization due to the need for the procedural treatment such as rigid bronchoscopy or flexible bronchoscopy. Foreign bodies are most frequently lodged in the right bronchi due to its vertical and wider compared to the left, followed by the larynx and trachea [19, 20]. Fluoroscopic guidance has emerged as a valuable tool for locating and removing foreign body, especially in pediatric patients [21, 22]. In uncommon cases, innovative techniques, such as using a bent flexible suction catheter to guide urological wire baskets during rigid bronchoscopy, have also been documented [23].

Delayed presentation to the ED, characterized in this study as a symptom onset of more than two hours, emerged as a significant predictor of hospitalization. Prolonged delays exacerbate complications, mirroring observations by previous study that delays longer than 24 h increase complication rates, lengthen surgical procedures, and extend hospital stays [24]. Late diagnoses are similarly linked to serious outcomes such as bronchiectasis, granulation tissue formation, and lung abscess [25]. These results underscore the necessity of prompt assessment and intervention to mitigate long-term respiratory harm and reduce the burden on healthcare resources.

Bronchoscopy is the primary diagnostic and therapeutic approach for FBA in children, with both flexible and rigid techniques used depending on the situation. Rigid bronchoscopy is often the modality of choice for removing foreign bodies due to its ability to secure the airway and facilitate precise manipulations [26]. Flexible bronchoscopy is increasingly employed as a first-line diagnostic tool, reducing the rate of negative rigid bronchoscopies [27, 28]. Early intervention by bystanders is associated with improved neurological outcomes [29]. Distally lodged metallic foreign bodies often necessitate specialized retrieval techniques. In our study, we successfully employed a 3 mm gold-plated neodymium magnet attached to flexible bronchoscopic forceps under fluoroscopic guidance to remove a metallic foreign body located in a very distal airway. To the best of our knowledge, this is the first reported case to use this combined approach, and our results were highly favorable. Previous research also demonstrated effective extraction of a metallic foreign body lodged in the right main bronchus using a magnet without fluoroscopic assistance [6]. Fluoroscopic guidance can be highly effective for retrieving airway foreign bodies in children, especially when they are situated in distal or otherwise difficult-to-access segments of the airways [21, 22]. Our study supports rigid bronchoscopy as the leading technique for managing pediatric FBA, largely due to its capacity for ventilation, airway control, and direct visualization, features that have established its status as the gold standard [14].

### Limitations

This study has several limitations that should be considered when interpreting its findings. First, its retrospective design relies on the accuracy and completeness of medical records, which may introduce information bias or limit the granularity of data on clinical presentation and outcomes. Second, as a single-center study conducted at a tertiary care hospital, the findings may not be generalizable to other healthcare settings, particularly those with fewer resources or differing demographic and socioeconomic profiles. Third, the study does not account for potential confounding factors such as variations in caregiver knowledge, access to timely medical care, or differences in procedural expertise, which may influence hospitalization rates and outcomes. One notable limitation of our study is the lack of detailed data on symptom onset and progression over time. As a result, we were unable to analyze the correlation between symptom evolution and the timing of diagnosis or intervention. This gap may have limited our ability to provide deeper insights into the natural history of FBA and its impact on treatment outcomes. Future prospective studies that include detailed symptom tracking and more detailed time stratifications are warranted to address this limitation. Additionally, the small number of hospitalized cases may limit the statistical power to detect associations with less common risk factors or interventions. Finally, while innovative techniques were explored in this study, their applicability and feasibility in other clinical environments remain uncertain, requiring further validation in larger, multicenter cohorts.

## Conclusions

This study underscores that younger age, delayed presentation to the ED, and foreign body location in the larynx-trachea-bronchus are significant predictors of hospitalization in pediatric FBA cases. Rigid bronchoscopy remains the primary treatment modality, while flexible bronchoscopy and innovative methods, such as magnet-assisted retrieval, offer effective solutions in specific scenarios. Early diagnosis and timely intervention are crucial to prevent complications, highlighting the need for increased clinical vigilance and caregiver education, particularly for high-risk age groups. These findings provide valuable insights for improving preventive strategies, optimizing management protocols, and exploring advanced techniques for FBA care in children.

## Data Availability

The datasets generated and/or analysed during the current study are not publicly available but are available from the corresponding author (RU) upon request.

## Abbreviations

STROBE: Strengthening the Reporting of Observational Studies in Epidemiology
SD: Standard deviation
IQR: Interquartile range
FBA: Foreign Body Aspiration
ED: Emergency Department
